# Agreement in breast lesion assessment and final BI-RADS classification between radial and meander-like breast ultrasound

**DOI:** 10.1186/s12880-021-00632-1

**Published:** 2021-06-22

**Authors:** Pascale Brasier-Lutz, Claudia Jäggi-Wickes, Sabine Schaedelin, Rosemarie Burian, Cora-Ann Schoenenberger, Rosanna Zanetti-Dällenbach

**Affiliations:** 1 Department of Obstetrics and Gynecology, University Hospital Basel, Spitalstrasse 21, 4056 Basel, Switzerland; 2 Department of Clinical Research, Statistics and Data Management, University Basel, Schanzenstrasse 55, 4031 Basel, Switzerland; 3 Department of Chemistry, University Basel, BioPark 1096, Mattenstrasse 24a, 4058 Basel, Switzerland; 4 Gynecology/Gynecologic Oncology, St. Claraspital Basel, Kleinriehenstrasse 30, 4085 Basel, Switzerland

**Keywords:** Agreement, BI-RADS final assessment, Breast ultrasound, Meander-like breast ultrasound, Radial breast ultrasound, Reliability

## Abstract

**Background:**

This study prospectively investigates the agreement between radial (r-US) and meander-like (m-US) breast ultrasound with regard to lesion location, lesion size, morphological characteristics and final BI-RADS classification of individual breast lesions.

**Methods:**

Each patient of a consecutive, unselected, mixed collective received a dual ultrasound examination.

**Results:**

The agreement between r-US and m-US for lesion location ranged from good (lesion to mammilla distance ICC 0.64; lesion to skin distance ICC 0.72) to substantial (clock-face localization κ 0.70). For lesion size the agreement was good (diameter ICC 0.72; volume ICC 0.69), for lesion margin and architectural distortion it was substantial (κ 0.68 and 0.70, respectively). Most importantly, there was a substantial agreement (κ 0.76) in the final BI-RADS classification between r-US and m-US.

**Conclusions:**

Our recent comparison of radial and meander-like breast US revealed that the diagnostic accuracy of the two scanning methods was comparable. In this study, we observe a high degree of agreement between m-US and r-US for the lesion description (location, size, morphology) and final BI-RADS classification. These findings corroborate that r-US is a suitable alternative to m-US in daily clinical practice.

*Trial registration*

NCT02358837. Registered January 2015, retrospectively registered https://clinicaltrials.gov/ct2/results?cond=&term=NCT02358837&cntry=&state=&city=&dist=

## Background

The majority of breast ultrasound (US) examinations involve a meander-like scanning procedure whereas radial breast ultrasound (r-US), also known as ductosonography, is typically applied only complementary to meander-like ultrasound (m-US) in case of nipple discharge [1] and to visualize intraductal pathologies [2], although a number of institutions and authors consider r-US a viable alternative to m-US [3–5]. However, to this day, r-US is not commonly used on its own in routine clinical practice although a wide transducer (92 mm) that allows for an efficient radial scanning of the breast is commercially available. As a result, there are only a small number of studies where breast US was performed by radial and not by meander-like scanning [6–10].

In breast ultrasound, the description of breast lesions is based on their sonographic features, and lesions are classified according to the Breast Imaging Reporting and Data System [11]. Most publications on the agreement of breast lesion description and BI-RADS classification are based on retrospective analysis of static images [12–25]. We are aware of only two studies [26, 27] that address the agreement of real time scanning of the same lesion between different examiners. To date, the agreement in describing and interpreting breast lesions in real time between radial breast ultrasound and meander-like ultrasound has not been investigated.

In a recent publication, we compared radial and meander-like scanning methods with regard to diagnostic accuracy and time used for the US examination [28]. The study revealed that the diagnostic accuracy for r-US and m-US is comparable as indicated by a sensitivity of 88.9% for both methods, a specificity of 86.4% for m-US and 89.4% for r-US, a positive predictive value of 64.0% for m-US and 69.6% for r-US, and a negative predictive value of 98.3% for both methods. Furthermore, we found a significantly shorter examination time for r-US (14.8 min) compared to m-US (22.6 min) supporting the notion that r-US is a viable alternative to m-US.

Given that the diagnostic accuracy of r-US equals that of m-US, we wanted to further explore whether r-US can be used as a stand-alone approach. Thus, we examined the agreement of the two scanning procedures with regard to the lesion location, the lesion size, the morphological characteristics and the final BI-RADS classification of individual breast lesions.

## Methods

From August 2011 to August 2014, we conducted this prospective single-center study (Department of Obstetrics and Gynecology, University Hospital Basel, Switzerland) which was approved by the local ethical committee (EKBB Nr. 123/11). To recruit women from an unselected, consecutive, mixed collective, a study information package was sent to all eligible subjects prior to the initial examination. All participating women signed the informed consent form. Study subjects were examined by meander-like and radial US on the same day by different examiners. The study population included asymptomatic women with either an increased risk for breast cancer or with dense breast tissue, symptomatic women presenting with breast pain or palpable breast lesions, and women with a history of breast cancer. We excluded men, women younger than 18 years of age, and women scheduled for minimal invasive breast biopsies.

Before the ultrasound examination, we recorded personal and family history, and performed a physical breast examination. All participants had a bilateral r-US and m-US in random order by different examiners who had access to the clinical findings, and, where available, to the mammographic results but not to the US examination of the other examiner.

All r-US were carried out by the same research fellow with limited experience in breast US who received a theoretical and practical didactic training in r-US at the onset of the study. M-US were performed by experts or beginners under the supervision of an expert, as it is common in teaching hospitals. All examiners received a yearly training in breast US.

The examiners used ultrasound equipment of the same type (EUB-7500 V 16–53 Step 3.5, Hitachi Medical Systems Europe Holding AG, Zug, Switzerland) for r-US and m-US examinations. A 50 mm wideband, high frequency (13–5 MHz) linear transducer (EUP-L74M; Hitachi Medical Systems Europe Holding AG, Zug, Switzerland) was employed for m-US. For r-US, a 92 mm wideband (10–5 MHz) linear transducer (EUP-L53L; Hitachi Medical Systems Europe Holding AG, Zug, Switzerland) was used with a water standoff (a water-filled latex cover) according to the manufacturer’s instructions (Hitachi Medical Systems Europe Holding AG, Zug, Switzerland). Both transducers had a center frequency of 7.5 MHz.

**Fig. 1:**
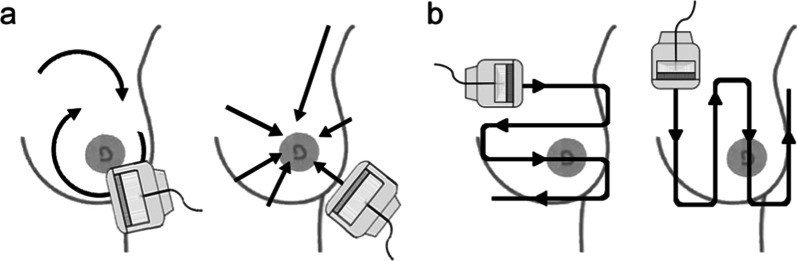
Radial and meander-like breast ultrasound. **a** Scheme of probe movement in radial scanning and in radial scanning of the axillary tail (left panel), and anti-radial movement (right panel). **b** Scheme of meander-like scanning movement in two orthogonal planes. Republished with adaptation from Arch Gynecol Obstet, from 'Comparison of radial and meander-like breast ultrasound with respect to diagnostic accuracy and examination time', Jäggi-Wickes et al., 301:1533, 2020; [18] with permission

Examiners saved an image with a timestamp at the beginning and at the end of each ultrasound examination to determine the duration of the examination. The US examinations were carried out as described in Jäggi et al. [28]. In brief, both types of breast US examination were conducted with the woman lying in an oblique supine position with her ipsilateral arm raised and her hand placed behind the head to flatten the breast tissue*.* As schematically illustrated in Fig. 1a, in r-US, the examiner moved the transducer first clockwise around the mammilla in a radial, and then in an anti-radial fashion, followed by a radial and anti-radial sweep of the upper outer quadrant to examine the axillary tail. In contrast, a meander- like scanning pattern was applied in vertical and transverse direction in m-US (Fig. 1b). Both r-US and m-US routinely included scanning of the axilla.

The examiners recorded the location of each lesion according to the clock-face. Based on the wide transducer in r-US, the mammilla is visualized as the rotation point, and thus, allowed for measuring the distance between lesion and nipple. In m-US, the nipple-lesion distance was estimated. In addition, in both US methods, the shortest distance between lesion and skin was recorded.

For m-US and r-US, we determined the dimensions of each sonographic lesion based on recordings in two orthogonal planes [28]. In addition, the morphologic features of each lesion were described. Examiners classified each lesion according to the BI-RADS Atlas [29]. Breast lesions classified as BI-RADS 4 or 5, and as BI-RADS 3 in patients with an increased breast cancer risk, were biopsied (sonographic-guided fine needle aspiration, core needle biopsy or vacuum-biopsy) for histological analysis.

Size, location, morphologic characteristics of the lesion and their final BI-RADS classification were electronically saved in the patient record (ViewPoint®, Version 5: GE Healthcare GmbH, Munich, Germany).

All data on patient and lesion characteristics extracted from the electronic patient records were entered into R (R Development Core Team 2018, Vienna, Austria) for data analysis.

### Statistical methods

Patient and lesion characteristics were summarized. Categorical data are presented as frequencies and percentages. For continuous variables, mean and standard deviation as well as range are presented.

In categorical variables, agreement between the two scanning procedures was quantified using κ-values with quadratic weights. However, for the endpoint “clock-face location” the cyclicity was taken into account by choosing weights according to the distance on the clock rather than absolute timepoints, meaning that the distance between "0" and "1" and between "11" and "0" is 1 h in both cases.

Weighted κ-values were interpreted as suggested by Landis et al. [30]: ≤ 0.20 poor agreement, 0.21–0.40 fair agreement, 0.41–0.60 moderate agreement, 0.61–0.80 substantial agreement, and 0.81–1.00 excellent agreement.

In continuous variables, the agreement was quantified using intraclass-correlation (ICC) [31]. The ICC is calculated based on analysis of variance. To this end, a mixed model is fitted to the data with scanning procedure and patient as random factors, and a fixed intercept was fitted. The ICC was estimated by dividing the variation related to the patient-to-patient difference by the total variance in the data. Therefore, ICC ranged between 0 and 1 and can be interpreted as the proportion of the variation of the data, which can be attributed to patient-to-patient variability. An ICC of 1 indicates a perfect agreement between r-US and m-US and that all differences in the ratings are due to differences in the patients. For the variable “mean volume”, the data was cube-root transformed prior to fitting the model since errors in main axes were inflated by the calculation of the volume leading to outliers not acceptable in the mixed model.

ICC-values were interpreted according to Cicchetti [32]: < 0.40 poor agreement, 0.40–0.59 fair agreement, 0.60–0.74 good agreement, and 0.75–1.00 excellent agreement.

## Results

We conducted 2327 dual ultrasound examinations of which 379 were excluded (18 examinations in patients younger than 18 years, 2 male patients, 56 incomplete US examinations, 128 incomplete informed consent forms, and 175 incomplete data sets). Consequently, 1984 dual ultrasound (US) examinations were analyzed. Among these, 168 suspicious lesions were detected in 148 patients (150 US examinations) (Table 1). We performed fine needle aspiration (n = 10, 6.0%), core needle biopsy (n = 146, 86.9%) or vacuum biopsy (n = 12, 7.1%) of all 168 suspicious lesions. Benign lesions were diagnosed in 132 (78.6%) cases while the other 36 (21.4%) were identified as breast cancer.

**Table 1:** Patient and lesion characteristics

Patient characteristics		Lesion characteristics	
Number of patients	148	Number of lesions	168
Positive personal history	3 (2.0%)	Benign lesions	132 (78.6%)
Positive family history	53 (35.8%)	Fibroadenoma	50
Breast cancer	43	Fibrosis/sclerosis	41
Ovarian cancer	2	Other B2 lesions	35
Breast and ovarian cancer	2	B3 lesions	6
Endometrial cancer	6	Malignant lesions	36 (21.4%)
Mean age in years	47.12	DCIS	2
(min, max) [SD]	(19–86) [± 14.73]	Invasive lobular cancer	3
		Invasive ductal cancer	31

The average age of patients with suspicious lesions was 47.1 years (19–86 years). Patients diagnosed with breast cancer (57.8 years) were significantly older (*p* < 0.01) than women with benign lesions (44.1 years). Three patients (2%) had a personal history of breast cancer and 53 (35.8%) a positive family history.

We analyzed the two scanning procedures with regard to their agreement in location, size, and morphologic characteristics of individual lesions, and the final BI-RADS classification of each breast lesion.

The lesion location was described by the clock-face localization, by its distance to the mammilla and to the skin. The values are presented in Table 2. The kappa-value of the clock-face localization for r-US and m-US was 0.70, indicating substantial agreement. The ICC-values of the two scanning methods for the distance from the lesion to the mammilla was 0.64, and 0.72 for the distance from the lesion to the skin, indicating good agreement.

**Table 2:** Agreement of lesion location

	Radial US^+^	Meander-like US^++^	ICC	Weighted kappa	Agreement
Clock-face localization				0.70	Substantial
Mean distance to mammilla (mm) (min, max) [SD]	28.6 (0.0–86.0) [± 20.5]	33.3 (0.0–100.0), [± 22.6]	0.64		Good
Mean distance to skin (mm) (min, max) [SD]	8.4 (1.0–26.0) [± 5.0]	6.9 (1.0–20.0) [± 4.0]	0.72		Good

^+^Due to the wide transducer in r-US, the mammilla is visualized as the rotation point, and thus, allowed for measuring the distance between lesion and nipple.^++^ In m-US, the nipple-lesion distance was estimated

The three dimensions of each lesion were determined in two orthogonal planes in r-US and m-US. The maximal diameter and the volume of each lesion obtained by either scanning method are presented in Table 3. Comparing the values revealed a good agreement (ICC 0.72 for lesion diameter and 0.69 for volume) between m-US and r-US.

**Table 3:** Agreement of lesion size

	Radial US	Meander-like US	ICC	Agreement
Mean max. lesion diameter (mm) (min, max) [SD]	14.6 (3.5–47.3) [± 8.7]	14.6 (3.4, 49.1) [± 8.9]	0.72	good
Mean volume [15] (min, max) [SD]	1.5 (0.01, 14.6) [± 2.6]	1.6 (0.01, 20.17) [± 3.1]	0.69	good

The morphological characteristics of each lesion were described according to the BI-RADS Atlas [29], and the final BI-RADS classification determined for r-US and m-US. The weighted kappa-values are presented in Table 4. Breast density, margin, architectural distortion showed an excellent or substantial agreement. Shape, posterior acoustic features, quality of assessment, orientation, and echo pattern showed moderate or fair agreement. The final BI-RADS classification substantially agreed (κ 0.76) between m-US and r-US.

**Table 4:** Agreement of morphological criteria and final BI-RADS assessment

	Weighted kappa	Agreement
Shape	0.47	moderate
Orientation	0.35	fair
Margin	0.68	substantial
Echo pattern	0.40	fair
Posterior acoustic features	0.47	moderate
Architectural distortion	0.70	substantial
Breast density*	0.81	excellent
Quality of assessment*	0.45	moderate
Final BI-RADS classification	0.76	substantial

^*^BI-RADS-Analogue[33]

## Discussion

We have recently shown that the diagnostic accuracy of radial scanning equals that of meander-like scanning in breast ultrasound [28]. Here, we examined the agreement between meander-like US and radial US with regard to lesion location, lesion size, morphologic characterization of breast lesions and the final BI-RADS assessment. Concerning the lesion location, our data demonstrate substantial agreement in the clock-face localization and good agreement for the distance from the lesion to the mammilla and to the skin. Lesion size shows good agreement between the two scanning procedures. The agreement of the different morphological features that characterize a breast lesion ranges from excellent to fair. Most importantly, the agreement of the final BI-RADS classification is substantial.

To the best of our knowledge, the agreement between m-US and r-US with regard to the parameters described above has not yet been investigated. A number of studies examine the agreement of the morphological features of breast lesions revealed by m-US examination and the final BI-RADS classification (see Table 5 and references therein). In contrast to the comparison of r-US and m-US presented in this study, the majority of these studies are based on the retrospective review of static images obtained by m-US. Real-time data acquisition was used only in a limited number of studies (Table 5) where different examiners perform m-US alone [26, 27].

**Table 5:** Literature comparison of BI-RADS agreement

		Final BI-RADS assessment	Shape	Orientation	Margin	Echo pattern	Posterior acoustic features
**Meander-like ultrasound versus radial ultrasound in real time**							
**This study**	2020	0.76	0.47	0.35	0.68	0.40	0.47
**Meander-like ultrasound versus meander-like ultrasound in real time**							
Yoon [26]	2011	0.37					
Berg [27]	2006	0.52	0.62	0.72	0.67	0.25	0.38
Berg ^#^ [34]	2006		0.14			0.61	0.45
**Meander-like ultrasound versus meander-like ultrasound by retrospective review of static images**							
Cho [25]	2019	0.49/0.52‡/0.63†					
Choi [12]	2018	0.70	0.67	0.63	0.55	0.57	0.60
Lee ҂ [24]	2016	0.04–0.59	0.17–0.60	0.25–0.77	0.05–0.52	0.05–0.55	0.33–0.64
Schwab [13]	2016	0.585–0.738					
Park [14]	2015	0.478	0.538	0.429	0.257	0.430	0.438
Elverici [15]	2013	0.35	0.45	0.66	0.33	0.41	0.54
Youk [16]	2013	0.38	0.61	0.62	0.39	0.54	0.57
Berg [17]	2012	0.53/0.59*	0.59	0.46	0.51	0.41	0.64
Cosgrove [18]	2012	0.59	0.58	0.53	0.38		
Schaefer [19]	2011	0.634					
Abdullah [20]	2009	0.3	0.64	0.70	0.36	0.58	0.47
Lee [21]	2008	0.53 / 0.62**	0,49	0.56	0.33	0.37	0.49
Park [22]	2007	0.49	0.42	0.61	0.32	0.36	0.53
Lazarus [23]	2006	0.28	0.66	0.61	0.40	0.29	0.40
**Meander-like ultrasound versus ABUS**							
Yun [35]	2019	0.61					
Vourtsis [36]	2018	0.99					
Barr [37]	2017	0.49					

^#^Phantom-Study

Improved final assessment after first‡, and second † quality workshop

҂Including κ values of faculty members, senior and junior residents

^*^Improved final assessment after feedback

^**^κ = 0.53 final BI-RADS assessment 3, 4a, 4b, 4c, 5 / κ = 0.62 final BI-RADS assessment 3, 4, 5

However, real-time examination is required for assessing the agreement of lesion location. Accordingly, Berg et al. [27] report an excellent agreement (ICC 0.84) in clock-face localization of breast lesions scanned by different examiners by m-US in real time. When we compare the clock-face localization of same breast lesion obtained by m-US and r-US, we observed a substantial agreement (weighted κ 0.70). With respect to the mean distance of the lesion to the mammilla, both studies reveal good agreement (ICC 0.71 and 0.64, respectively). However, neither Berg et al. nor any other study that we are aware of evaluated the mean distance from the lesion to the skin, which shows a good agreement (ICC 0.72) between m-US and r-US.

In addition, real-time scanning allowed us to assess the agreement of the lesion size. The agreement in the mean lesion diameter was excellent (ICC 0.87) in m-US versus r-US [27] and good (ICC 0.72) in m-US versus r-US (this study). Moreover, we do not know of any other study addressing the agreement of the mean lesion volume where we find a good agreement (ICC 0.69) between m-US and r-US.

The agreement for m-US and r-US in the morphological assessment of breast lesions ranged from fair (for orientation and echo pattern) to moderate (for shape and posterior acoustic features) and substantial (for margin and architectural distortion). In comparison to Berg and colleagues [27] who investigated the agreement between different examiners performing real-time m-US of the same lesion, our κ-values for the agreement between r-US and m-US are higher for echo pattern and posterior acoustic features, similar for margin, and lower for shape and orientation. In another study on m-US using a phantom [34], κ-values were lower for shape, higher for echo pattern and similar for posterior acoustic features compared to our data (Table 5). We conclude that the overall agreement in real-time between r-US and m-US is similar to that of m-US alone. This conclusion is further corroborated by comparing our data for both real-time scanning methods to published data obtained by retrospective review of static images performed by m-US (Table 5).

Most importantly, we find a substantial agreement in the final BI-RADS assessment between r-US and m-US (κ 0.76). The k value is not only higher than those reported for real-time m-US but also higher than most κ values found for BI-RADS agreement in studies retrospectively reviewing static images (Table 5). Comparing final BI-RADS assessment in automated breast ultrasound (ABUS) and m-US agreement k values of 0.61[35] and 0.49 [37] in a selected study population, and k value of 0.99 [36] in a screening situation have been reported. In daily clinical practice, the management of patients with breast disease is largely determined by the final BI-RADS classification [38]. Thus, a substantial agreement in final BI-RADS classification between m-US and r-US is prerequisite for the validation of r-US as an alternative scanning procedure.

There are a number of limitations associated with the comparison of m-US and r-US in this study. As we recently reported for diagnostic accuracy [28] not all patients agreed to participate in the study, conceivably owing the time requirement of a second ultrasound examination. Thus, the study collective may not fully represent the consecutive, mixed population of an outpatient breast clinic. The dataset analyzed takes only a limited number of BI-RADS 3 lesions into account. BI-RADS 3 lesions with no additional risk factors and BI-RADS 2 lesions are generally not biopsied, and were therefore not included in this study due to the lack of a confirming histology. Furthermore, the difference in transducer width in r-US (92 mm) versus m-US (50 mm) represents a technical limitation in so far that the distance from the lesion to the mammilla is measured in r-US but estimated in m-US.

Real-time assessment does not allow for m-US and r-US to be carried out by the same examiner which could be considered a limiting factor. However, in actual practice the evaluation of US features is performed in real time during the US examination and thus, this study reflects routine clinical settings. Moreover, as is common for teaching hospitals, ultrasound was in some cases performed by examiners with less experience which, at first sight, appears to be a limitation. However, these examiners were always supervised by an expert and therefore, the quality of data acquisition was not influenced by different educated examiners.

## Conclusion

The agreement of lesion description (location, size, morphology) and final BI-RADS classification between meander-like and radial breast ultrasound are good and substantial. Taking into account that also the diagnostic accuracy between the two scanning methods is comparable, radial breast ultrasound can be considered a suitable alternative to meander-like breast ultrasound in daily clinical practice.

## Abbreviations

r-US: Radial breast ultrasound
m-US: Meander-like breast ultrasound
US: Breast ultrasound
ICC: Intraclass-correlation
ABUS: Automated breast ultrasound

## Declaration

### Availability of data and materials

The datasets used and analyzed during the current study are available from the corresponding author on reasonable request.
